# Influence of Handrim Wheelchair Propulsion Training in Adolescent Wheelchair Users, A Pilot Study

**DOI:** 10.3389/fbioe.2015.00068

**Published:** 2015-05-18

**Authors:** Jennifer L. Dysterheft, Ian M. Rice, Laura A. Rice

**Affiliations:** ^1^Wheelchair Biomechanics Performance Laboratory, Department of Kinesiology and Community Health, University of Illinois Urbana-Champaign, Urbana, IL, USA

**Keywords:** manual wheelchair, propulsion, biomechanics, training, adolescents

## Abstract

Ten full-time adolescent wheelchair users (ages 13–18) completed a total of three propulsion trials on carpet and tile surfaces, at a self-selected velocity, and on a concrete surface, at a controlled velocity. All trials were performed in their personal wheelchair with force and moment sensing wheels attached bilaterally. The first two trials on each surface were used as pre-intervention control trials. The third trial was performed after receiving training on proper propulsion technique. Peak resultant force, contact angle, stroke frequency, and velocity were recorded during all trials for primary analysis. Carpet and tile trials resulted in significant increases in contact angle and peak total force with decreased stroke frequency after training. During the velocity controlled trials on concrete, significant increases in contact angle occurred, as well as decreases in stroke frequency after training. Overall, the use of a training video and verbal feedback may help to improve short-term propulsion technique in adolescent wheelchair users and decrease the risk of developing upper limb pain and injury.

## Introduction

Manual wheelchair propulsion (MWP) for daily mobility places significant demands on the upper limbs. While performing everyday tasks, such as propulsion and transferring, manual wheelchair users (MWUs) repetitively experience large loads through the shoulders and wrists (Bayley et al., 1987; Nash et al., 2001). As a consequence, MWUs experience disproportionately high rates of overuse injury and pain (Burnham and Steadward, 1994; Curtis et al., 1995; Ballinger et al., 2000). For example, nearly 70% of individuals who regularly use a manual wheelchair will experience upper limb pain, at the wrists or shoulders (Bayley et al., 1987; Gellman et al., 1988; Wylie and Chakera, 1988; Burnham and Steadward, 1994; Rice et al., 2013). The consequences of overuse injuries and pain may greatly impact MWUs’ functional capacity and mobility, negatively influencing independence and quality of life (Gutierrez et al., 2007).

With upper limb pain and injury becoming increasingly common, the Consortium for Spinal Cord Medicine (CSCM) has recommended that MWUs use a low frequency, long and smooth stroke during the propulsive phase to decrease force exerted at a given velocity while allowing the hand to drift down and back below the handrim during recovery (Consortium for Spinal Cord Medicine, 2005). These recommendations are meant to minimize task repetition as well as the magnitude of propulsive forces through use of a larger contact angle (Boninger et al., 2005; Medicine PVoACfSC, 2005). Contact angle is the angle along the arc of the handrim, from contact to release. A larger *Contact Angle* is recommended, as it has the potential to reduce the number of strokes needed to maintain a given speed, therefore reducing *Stroke Frequency* and the number of repetitive motions performed by the upper limbs. Additionally, *Peak Resultant Force* is the occurrence of the highest vector sum of component forces (Fx, Fy, Fz) applied to the handrim during propulsion. Elevated peak forces experienced at the shoulder during propulsion often contribute to joint damage and overuse injuries as well (Shimada et al., 1998; Nyland et al., 2000; Vanlandewijck et al., 2001). Therefore, it is reasonable to surmise that utilizing techniques to minimize peak forces may help reduce the risk of pain and injury development.

Although these recommendations are well documented, alarmingly, children who use manual wheelchairs rarely receive formal training on safe and effective wheelchair propulsion techniques (Sawatzky et al., 2012). Lack of training may heighten the risk of injury development; however, training interventions have successfully improved propulsion technique in adult MWU (Rice et al., 2010, 2013). Most importantly, these studies produced substantial positive changes in contact angle, stroke frequency, and peak forces with video training and verbal feedback (Rice et al., 2010, 2013). While the literature on adult propulsion biomechanics and training is well developed, few have explored technique modification strategies in adolescent MWUs. Although basic skill and resistance training strategies have produced some positive results in adolescent MWUs (O’Connell and Barnhart, 1995; Sawatzky et al., 2012), it remains unclear if children can benefit from training approaches, proven successful in adults. If propulsion mechanics can be improved early in life, prior to technique consolidation, it may set adolescent MWUs on a healthy trajectory into adulthood.

The purpose of this study was to examine the safety and effectiveness of a propulsion technique training system, in adolescent MWU’s, which has been used previously to successfully train adults. The system is a practical approach based on instructional video and verbal feedback. The goal of training was to instruct adolescent users to maximize contact angle, while minimizing stroke frequency at the handrim (Rice et al., 2010, 2013). If successful, the training system will represent a low-cost practical approach to minimizing upper limb pain and injury development in adolescent MWU’s. Additionally, results may help to determine if adolescent wheelchair users display stroke mechanics changes similar to those seen in adults. Based on previous literature, it was hypothesized that with training, adolescents would react similarly to adults, displaying increased contact angle with reductions in stroke frequency and peak force at the handrim.

## Materials and Methods

### Participants

The University of Illinois at Urbana-Champaign’s Institutional Review Board (IRB) approved all procedures prior to experimentation. Informed consent was attained from parents while assent was gathered from adolescent study participants. Inclusion criteria specified for participation included individuals 8–18 years of age who independently propelled a manual wheelchair as their primary mode of mobility (>40 h/week of wheelchair use). Additionally, all participants were free of any upper extremity condition or disability that could be worsened by physical activity and, participants with a spinal cord injury >2 years post-injury. A convenience sample of 10 adolescents (7 male, 3 female; 15.8 ± 1.6 years) recruited from the University of Illinois Youth Wheelchair Skills Camp, volunteered to participate in the study. Participant demographics are presented in Table 1.

**Table 1:** **Participant demographics**.

Participant ID	Age	Gender	Diagnosis/injury level	Wheelchair use	Years using manual chair	WUSPI pre-intervention	WUSPI post-intervention
1	16	M	SB	Full time	16	6.4	6
2	15	M	CMT	Full time	9	0	SNR
3	14	F	SCI (C7)	Full time	10	23.1	4.1
4	13	F	SB	Full time	12	1.8	0
5	15	F	Amp	Full time	10	0.4	0
6	17	M	SB	Full time	6	22.8	26
7	17	M	SB	Full time	15	6	2.6
8	18	M	SCI	Full time	3	3.1	SNR
9	17	M	SCI (T5)	Full time	10	35.5	SNR
10	16	M	SB	Full time	16	0	0

*SB, spina bifida; SCI, spinal cord injury (injury level); Amp, amputee; CMT, Charcot–Marie–Tooth disease; SNR, post-intervention survey not returned (no scores)*.

### Equipment

**Figure 1:**
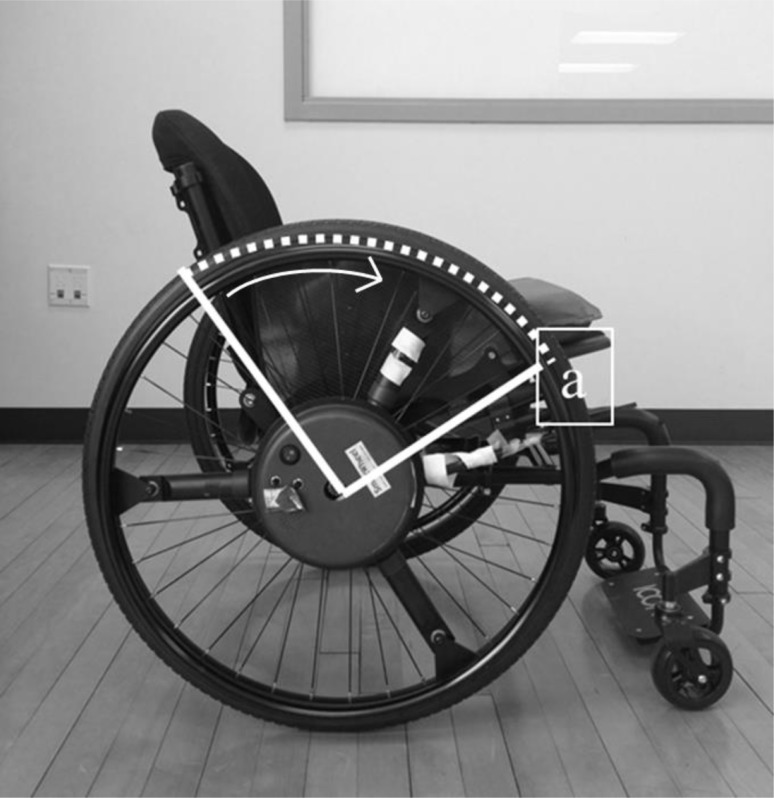
**Clinical SMARTwheel**. Clinical SMARTwheels used for data collection replaced both wheels on participants’ personal chairs. ^a^denotes training intervention emphasis of using a large contact angle, along with decreased stroke frequency and forces.

For data collection, force and moment sensing Clinical SMARTwheels (SMARTwheel; Three Rivers Holdings, Mesa, AZ, USA) (Asato et al., 1993) were fitted bilaterally to replace both wheels on the participants’ personal, everyday wheelchairs (Figure 1). The right SMARTwheel was used for data collection, while the left served as a dummy wheel to parallel weight and inertial characteristics. While the SMARTwheel is slightly heavier than a standard wheel, it does not alter the feel or setup of a participant’s personal chair (Cooper et al., 1997).

### Protocol

#### Pain Assessment

First, participants completed the wheelchair users shoulder pain index (WUSPI) survey. The WUSPI (Curtis et al., 1995, 1999), a reliable and valid 15-item questionnaire was used to quantify the current level of pain in all participants (Curtis et al., 1995). Participants completed the WUSPI prior to propulsion activities. Additionally, the tool was sent to participants 3 months after data collection to examine the possible influence of training on shoulder pain (the extent to which pain resulted from or was worsened by participation in the study).

#### Propulsion Assessment

**Figure 2:**
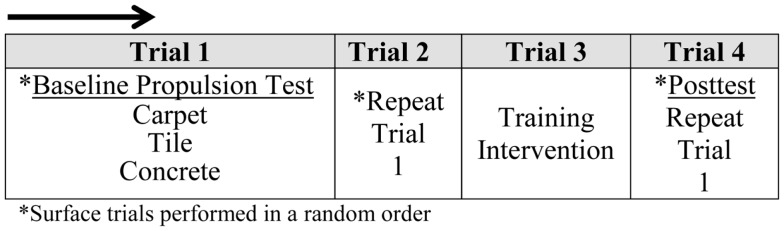
**Experimental study design**.

With SMARTwheels, participants were instructed to push at a natural, self-selected pace over flat, industrial grade carpeted, and tiled surfaces, two times each (Figure 2). A self-selected speed was chosen deliberately to examine propulsion mechanics occurring at natural and comfortable pace, as well as to maximize the safety of adolescent participants. Participants also performed two speed controlled trials over a flat concrete surface. Based on the self-selected speeds from existing literature, a target velocity of 1.5 m/s was selected (Van der Woude et al., 1986; Bednarczyk and Sanderson, 1995). This pace is slightly slower than previous studies to ensure all participants could safely and comfortably maintain the speed. Participants were instructed to match the speed of a researcher using a wheelchair equipped with a Garmin Edge 800 GPS Speedometer.

All trials/surfaces were completed in random order for a distance of 15 m to allow completion of five steady-state strokes. Rest periods were not needed between surface trial repeats due to the low intensity and short distances; however, 5 min of recovery were provided in the time needed to change between surfaces. Additionally, researchers provided no feedback or commentary on propulsion mechanics or techniques during these trials as they occurred prior to training. After participants were exposed to the intervention (described below), propulsion was evaluated on each surface one additional time. Data were collected during the entire propulsion period for all trials on each surface. Due to the small sample size, participants served as their own control, where trials one and two constituted baseline data collection used for comparison with the third, post-intervention trial.

### Intervention

After participants completed the first two trials over all three surfaces, they participated in the training intervention. The intervention consisted of a short training video (5 min) (Rice et al., 2013), which allowed for independent viewing. Participants were encouraged to use low frequency, long and smooth strokes (large contact angle) during the propulsive phase to decrease force exerted at a given velocity (Consortium for Spinal Cord Medicine, 2005). Additionally, subjects were encouraged to match the speed of the handrim upon contact to minimize braking torques that slows the wheel. Both contact angle and stroke frequency were defined and discussed in the video. As a visual aid, the video contained a MWU propelling on a dynamometer, demonstrating these techniques. For additional motivation, the video emphasized the importance of using proper technique to preserve upper limb health, independence, and quality of life. Upon completion of the video, researchers discussed and reemphasized the primary components of the video. During this time, participants were given the opportunity to ask researchers any questions and practice the new propulsion techniques. During this practice, participants received basic feedback from researchers.

### Data reduction

The propulsion performance variables selected for analysis were peak resultant force (Newton), contact angle (Degree), and stroke frequency (strokes per second) because of their association with upper limb pain and injury development. Participants’ average velocity (m/s) was recorded for each trial as well. Mean intra-individual variability was calculated for each performance variable during velocity controlled concrete trials to quantify potential motor learning strategies as a function of training. Coefficient of variation (CV) was only calculated for the speed controlled trials to minimize the potential occurrence of variation changes due to velocity fluctuations. CV (%) was calculated as the percentage of SD with respect to the mean. Data from the Clinical SMARTwheel were collected from forces and moments applied to the handrim at a sampling frequency of 240 Hz. All variables were calculated as the mean values of five steady-state strokes.

### Statistical analysis

All statistical analyses were performed using SPSS (v.20.0 SPSS Inc., Chicago, IL, USA). Differences in normally distributed propulsion and intra-individual variation variables during the trials were analyzed separately using multiple one-way repeated measures ANOVAs with Bonferroni adjusted *post hoc* testing. Variables that violated the Shapiro–Wilk test of normality (*p* < 0.05) were analyzed using non-parametric Freidman’s tests with Bonferroni corrections for pairwise comparisons. To examine possible effects or changes of speed, a one-way repeated measures ANOVA was performed on the average velocity of each trial. The first two, pre-intervention trials were analyzed separately to better differentiate effects of the intervention from possible practice effects that may have occurred due to repetition and practice (De Groot et al., 2002, 2008; Vegter et al., 2014). All variable analysis was performed separately for each surface. All WUSPI data from the pre-intervention and 3 month post-intervention follow-up (*n* = 7) were compared using a paired samples *t*-test.

The criterion to reject the null hypothesis was *p* < 0.05 and sample effect sizes are interpreted as small (η^2^ < 0.20), moderate (η^2^ ~ 0.50), and large (η^2^ > 0.80). All descriptive statistics are reported as Mean (Standard Deviation) [M(SD)].

## Results

### Propulsion performance

Descriptive statistics are reported in Tables 2–4. Of the variables from the carpet trials (Table 2), stroke frequency (trial 1: *p* < 0.01) violated the Shapiro–Wilk test of normality and was therefore analyzed using Freidman’s tests. Results indicated that during the carpeted trials, after the intervention, statistically significant increases occurred in peak resultant force (*p* < 0.01, η^2^ = 0.48) and contact angle (*p* = 0.04, η^2^ = 0.30) with decreased stroke frequency (*p* = 0.048, η^2^ = 0.22). Separate analysis of the average velocity for each of the trials revealed significant differences in speed during the self-selected trials (*p* = 0.02, η^2^ = 0.35).

**Table 2:** **Carpet propulsion trials**.

Performance variable	Trial 1 *M* (SD)	Trial 2 *M* (SD)	Trial 3 *M* (SD)	*F*	ω^2^
**Parametric results**					
Peak resultant force (N)	49.96 (16.67)	51.90 (14.0)	60.99 (18.36)	8.19*	0.32
Contact angle (Deg)	71.29 (19.22)	78.80 (21.45)	82.66 (16.38)	3.80*	0.16
Velocity (m/s)	1.12 (0.21)	1.17 (0.15)	1.33 (0.28)	4.83*	0.35
**Non-parametric results**				***X*^2^**
Stroke frequency (stroke/s)	0.82 (0.09)	0.84 (0.10)	0.76 (0.12)	6.05*	0.09

*p < 0.05.

**Table 3:** **Tile propulsion trials**.

Performance variable	Trial 1 *M* (SD)	Trial 2 *M* (SD)	Trial 3 *M* (SD)	*F*	ω^2^
**Parametric results**					
Contact angle (Deg)	67.90 (18.68)	73.94 (21.20)	81.28 (18.59)	4.60*	0.19
Velocity (m/s)	1.07 (0.10)	1.08 (0.13)	1.14 (0.17)	1.37	0.13
**Non-parametric results**				***X*^2^**
Peak resultant force (N)	46.95 (15.68)	46.37 (9.90)	59.37 (19.43)	7.40*	0.29
Stroke frequency (stroke/s)	0.80 (0.08)	0.80 (0.11)	0.72 (0.08)	7.32*	0.08

*p < 0.05.

**Table 4:** **Outdoor propulsion trials**.

Performance variable	Trial 1 *M* (SD)	Trial 2 *M* (SD)	Trial 3 *M* (SD)	*F*	ω^2^
**Parametric results**					
Contact angle (Deg)	69.28 (16.73)	72.26 (12.66)	84.13 (17.77)	5.14*	0.22
Peak resultant force (N)	59.85 (21.08)	51.19 (15.80)	67.10 (26.59)	3.45	0.14
Stroke frequency (stroke/s)	0.94 (0.08)	0.87 (0.09)	0.75 (0.16)	5.71*	0.24
Velocity (m/s)	1.46 (0.07)	1.46 (0.06)	1.45 (0.07)	0.06	0.03

*p < 0.05.

From the tile trials (see Table 3), peak resultant force (trial 1: *p* = 0.02) and stroke frequency (trial 2: *p* < 0.01) violated the Shapiro–Wilk test of normality and were therefore analyzed using Freidman’s tests. Results of the tile trials indicated that after the intervention, statistically significant increases in contact angle (*p* = 0.02, η^2^ = 0.34) and peak resultant force (*p* = 0.03, η^2^ = 0.44) occurred, as well as a significantly decreased stroke frequency (*p* = 0.03, η^2^ = 0.22). Differences in velocity were not found to be statistically significant between the tile trials (*p* = 0.28, η^2^ = 0.13).

No variables from the speed controlled concrete trials violated the Shapiro–Wilk test of normality. Results (Table 4) demonstrate statistically significant increases in contact angle (*p* = 0.02, η^2^ = 0.36) with decreased in stroke frequency (*p * = 0.04, η^2^ = 0.39) after training. A trend was observed for increases in peak force (*p * = 0.05, η^2^ = 0.28). No significant differences were observed in average velocity between trials (*p* = 0.54, η^2^ = 0.07).

### Intra-individual variability performance

Dependent variables from the velocity controlled concrete trials were analyzed with a one-way repeated-measure ANOVA. Statistically significant changes were found only in peak resultant force variation (*p * = 0.02, η^2^ = 0.35) (Table 5). No other significant changes were observed in contact angle (*p* = 0.30, η^2^ = 0.12), stroke frequency (*p* = 0.32, η^2^ = 0.12), and velocity (*p* = 0.84, η^2^ = 0.20).

**Table 5:** **Outdoor propulsion trials: variability results**.

Performance variable	Trial 1 *M* (SD)	Trial 2 *M* (SD)	Trial 3 *M* (SD)	*F*	ω^2^
**Parametric results**					
Contact angle (Deg)	18.93 (9.97)	19.18 (10.74)	12.97 (9.03)	1.31	0.02
Peak resultant force (N)	17.79 (8.66)	24.81 (8.11)	16.86 (8.68)*	4.76	0.20
Stroke frequency (stroke/s)	13.58 (11.41)	19.40 (10.22)	14.75 (11.36)	1.20	0.01
Velocity (m/s)	4.30 (2.60)	4.03 (2.60)	3.49 (2.04)	0.18	0.06

*p < 0.05.

### Shoulder pain

Results of the paired samples *t*-test revealed no significant change in WUSPI scores from pre-testing (8.64 [10.09]) to the 3 month follow-up (5.53 [9.32]), *t*(6) = 1.13, *p * = 0.30 (Table 1). Three participants did not return the WUSPI, 3 months post-intervention (*n* = 7).

## Discussion

The purpose of this study was to examine the influence of wheelchair training on adolescent propulsion technique to reduce the risk of upper limb pain and injury development. Because few studies have implemented training protocols on adolescent wheelchair users, another goal of the study was to determine if adolescents demonstrated propulsion technique changes similar to those seen in adult wheelchair users. Consistent with our hypothesis, changes occurred in participants’ propulsion technique following the intervention, similar to those found in adult wheelchair users (Rice et al., 2010, 2013). Specifically, participants demonstrated increased contact angle, with decreases in stroke frequency across all surfaces and speeds, with moderate effect sizes. Additionally, when velocity was controlled a trend toward increased peak force was observed, however, small in magnitude.

**Figure 3:**
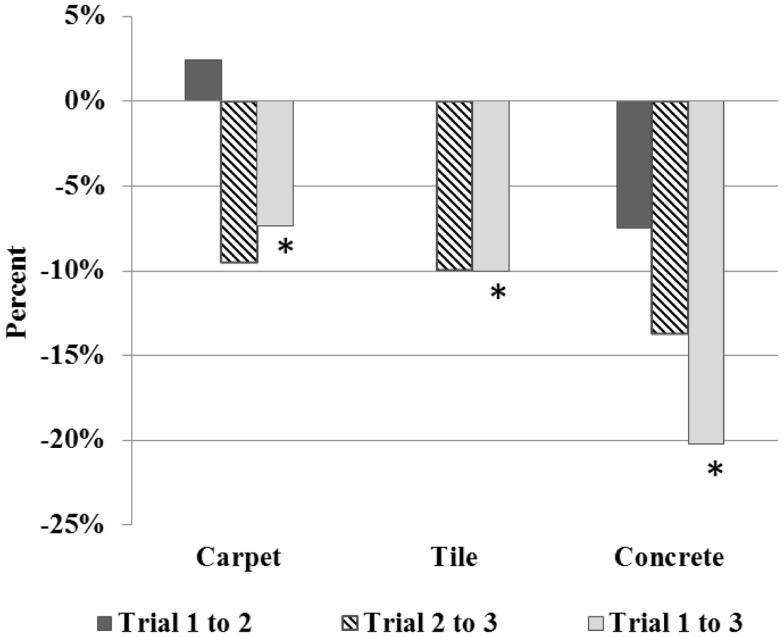
**Percentage change in stroke frequency over trials**. *denotes significant change between trials, *p* < 0.05.

**Figure 4:**
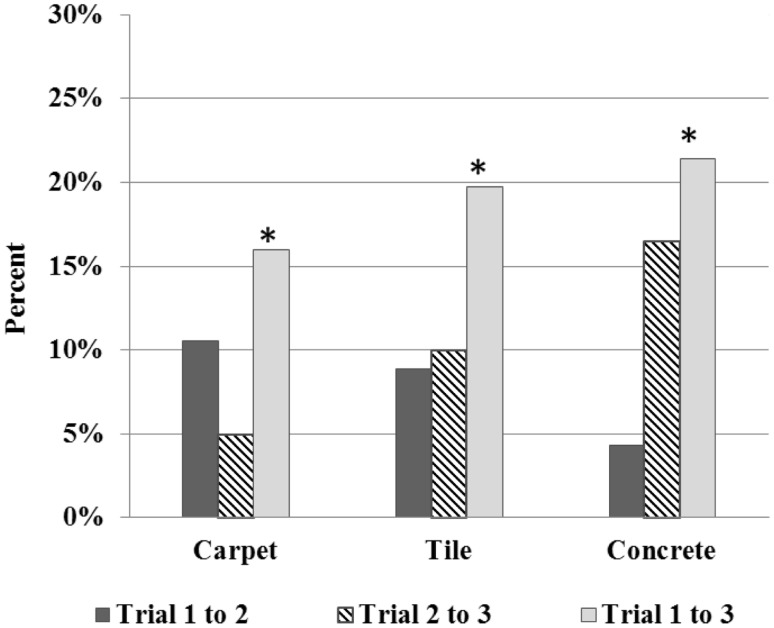
**Percentage change in contact angle over trials**. *denotes significant change between trials, *p* < 0.05.

During both the carpet and tile trials, significant increases were observed in contact angle, which likely resulted in participants’ significant decrease in stroke frequency (see Figures 3–4). Although more recent literature on adults has observed decreases in force application with training, increases in peak forces occurred during the carpet and tile trials in the current study. In the study by Rice et al. (2013), similar short-term increases in force occurred; however, with further time and training, participants peak force levels subsided 3 months later. As previously observed in adults, the use of a larger contact angle may lead to a stroke where forces are distributed over a greater angular distance of the handrim. Based on the short-term similarities observed between adolescents in the current study and adults (increased short-term force application), it is possible that adolescent MWU may too learn to apply less peak force utilizing a larger contact angle. Long-term propulsion technique follow-up has been scheduled to help clarify. Additionally, use of larger contact angle helped minimize stroke frequency to maintain similar speeds immediately in the current study supporting the protective ergonomic principle of task reduction (Boninger et al., 2000; Medicine PVoACfSC, 2005). Overall, the observed short-term similarities between previous adult literature and our adolescents may provide preliminary evidence children can benefit from similar training approaches.

Although the increased forces observed in the current study were small in magnitude, it is important to discuss their etiology due to the association with upper limb pathologies. A likely explanation for the increased forces occurring on carpet was due to increased average speeds (Tables 2 and 3). As observed in the concrete trials, when velocity was regulated, peak force remained relatively stable post-intervention, with only a slight trend toward a gain (Table 4). Additionally, it is important to note that although no intervention had taken place yet, notable differences in peak force occurred during the two initial concrete control trials (Table 4). Numerous factors may have played a role in these peak force changes, such as attempts to maintain a target speed, adaptation to the research environment, or natural variation of force application. It is possible that with additional trials, changes in peak force may have minimized from one trial to the next. However, for the sample population in general, these changes in performance suggest that further research is needed to examine performance consistency.

Intra-individual variability of the propulsion variables were found to be relatively stable over all three surfaces, both prior (trials 1–2) to and following training (trial 3). However, this may be a result of the very short time-period, and therefore the limited number of strokes, from which data were collected. In previous literature examining variability in MWP, data were collected for 3–12 min for each trial, allowing for an extensive number of propulsive strokes to be analyzed (Moon et al., 2013; Vegter et al., 2013, 2014). When learning a new skill, it is anticipated that reductions in variability may occur with time. As observed previously in adults, the amount of variability in propulsion mechanics decrease more quickly in individuals who were considered to be faster learners (Bartlett et al., 2007; Wang et al., 2012; Vegter et al., 2013). However, it is also believed that a lack of variation in any repetitive movement results in an insufficient amount of time for the limbs to adapt or heal, thus resulting in overuse injuries (Bartlett et al., 2007). As individuals modify their movements slightly altering the distribution of movement stresses from one repetition to the next, a particular range of variability may serve as a protective measure against overuse injuries (Madeleine et al., 2008). Further research into the variability of both adolescent and adult wheelchair users may allow researchers to ascertain if a particular range is favorable.

Although not found to be statistically significant, baseline WUSPI scores revealed our participants had relatively low levels of shoulder pain prior to training and further reduced pain 3 months later (see Table 1). Importantly, due to our study design, we cannot conclude the pain reduction observed was directly related to our intervention. It is also possible the 3-month follow-up was biased because three individuals did not return their surveys. Future studies should explore pain reduction as a function of propulsion technique modification in larger groups of wheelchair users over time.

Although significant changes were found in contact angle and stroke frequency, the changes may have been modest in comparison to a less active population. However, even with wheelchair athletics experience, our participants demonstrated room for improvement. The literature supports that experienced MWUs have been observed to use larger contact angles and lower peak forces in comparison to non-experienced groups (Robertson et al., 1996; Kotajarvi et al., 2004).

As we move forward with this line of research, it will be critical to include larger more diverse groups of younger MWUs to both maximize generalizability and to sufficiently power future investigations. Additionally, we plan to shift outcome measures away from the short-term influence of training observed in a laboratory setting to the long-term effects occurring at home and in the community. Use of minimally obtrusive technologies like accelerometers has enormous potential in this context. For example, vector counts accumulated though wrist worn accelerometers are shown to have a linear association with energy expenditure during propulsion (Learmonth et al., 2015) and may offer a more detailed account of how improved technique translates to physical activity.

### Limitations

As the current study was one of the first pilot investigations of propulsion training in adolescent wheelchair users, numerous limitations exist. Obtaining a sufficiently large sample size of younger wheelchair users can be challenging and likely explains the lack of literature on the topic. Similarly, our small, relatively homogenous sample size of active wheelchair users was a significant limitation, which influenced our statistical approach. Consequently, a repeated measures design was implemented where individuals served as their own control, which may have reduced power, as well as inflating the possibility of Type II error. Additionally, because no long-term data were collected, it is not possible to determine if learned propulsion techniques would persist or if reductions in peak force would eventually occur. Additionally, wheelchair characteristics were not collected, which may have influenced technique modification. Finally, the small number of strokes analyzed may have decreased our ability to detect fluctuations in intra-individual variation. Future examination will include longer periods of data collection to confirm.

## Conclusion

After a 5 min training video, adolescent wheelchair users experience significant positive changes in contact angle and stroke frequency similar to those seen in adults, which may prevent the development of upper limb pain and injury. Although short-term changes were similar to those seen in adults, future investigation is warranted on larger more age divers group of younger MWU to confirm differences with adults.

## Conflict of Interest Statement

The authors declare that the research was conducted in the absence of any commercial or financial relationships that could be construed as a potential conflict of interest.

## References

[B1] Asato K. T. Cooper R. A. Robertson R. N. Ster J. F. (1993). SMARTWheels: development and testing of a system for measuring manual wheelchair propulsion dynamics. IEEE Trans. Biomed. Eng. 40, 1320–1324. 10.1109/10.250587 8125507

[B2] Ballinger D. A. Rintala D. H. Hart K. A. (2000). The relation of shoulder pain and range-of-motion problems to functional limitations, disability, and perceived health of men with spinal cord injury: a multifaceted longitudinal study. Arch. Phys. Med. Rehabil. 81, 1575–1581. 10.1053/apmr.2000.18216 11128892

[B3] Bartlett R. Wheat J. Robins M. (2007). Is movement variability important for sports biomechanists? Sports Biomech. 6, 224–243. 10.1080/14763140701322994 17892098

[B4] Bayley J. C. Cochran T. Sledge C. (1987). The weight-bearing shoulder. The impingement syndrome in paraplegics. J. Bone Joint Surg. 69, 676–678. 3597466

[B5] Bednarczyk J. H. Sanderson D. J. (1995). Limitations of kinematics in the assessment of wheelchair propulsion in adults and children with spinal cord injury. Phys. Ther. 75, 281–289. 7899486 10.1093/ptj/75.4.281

[B6] Boninger M. L. Baldwin M. Cooper R. A. Koontz A. Chan L. (2000). Manual wheelchair pushrim biomechanics and axle position. Arch. Phys. Med. Rehabil. 81, 608–613. 10.1016/S0003-9993(00)90043-1 10807100

[B7] Boninger M. L. Koontz A. M. Sisto S. A. Dyson-Hudson T. A. Chang M. Price R. (2005). Pushrim biomechanics and injury prevention in spinal cord injury: recommendations based on CULP-SCI investigations. J. Rehabil. Res. Dev. 42, 9. 10.1682/JRRD.2004.08.0103 16195959

[B8] Burnham R. S. Steadward R. D. (1994). Upper extremity peripheral nerve entrapments among wheelchair athletes: prevalence, location, and risk factors. Arch. Phys. Med. Rehabil. 75, 519–524. 8185443

[B9] Consortium for Spinal Cord Medicine. (2005). Preservation of Upper Limb Function Following Spinal Cord Injury: A Clinical Practice Guideline for Health-Care Professionals. Washington, DC: Paralyzed Veterans of America. 10.1080/10790268.2005.11753844 PMC1808273 16869091

[B10] Cooper R. A. Robertson R. N. VanSickle D. P. Boninger M. L. Shimada S. D. (1997). Methods for determining three-dimensional wheelchair pushrim forces and moments - a technical note. J. Rehabil. Res. Dev. 34, 162–170. 9108343

[B11] Curtis K. Roach K. Applegate E. B. Amar T. Benbow C. S. Genecco T. D. (1995). Development of the wheelchair user’s shoulder pain index (WUSPI). Spinal Cord 33, 290–293. 10.1038/sc.1995.65 7630657

[B12] Curtis K. A. Drysdale G. A. Lanza R. D. Kolber M. Vitolo R. S. West R. (1999). Shoulder pain in wheelchair users with tetraplegia and paraplegia. Arch. Phys. Med. Rehabil. 80, 453–457. 10.1016/S0003-9993(99)90285-X 10206610

[B13] De Groot S. De Bruin M. Noomen S. Van der Woude L. (2008). Mechanical efficiency and propulsion technique after 7 weeks of low-intensity wheelchair training. Clin. Biomech. 23, 434–441. 10.1016/j.clinbiomech.2007.11.001 18077065

[B14] De Groot S. Veeger D. Hollander A. P. Van der Woude L. (2002). Wheelchair propulsion technique and mechanical efficiency after 3 wk of practice. Med. Sci. Sports Exerc. 34, 756–766. 10.1097/00005768-200205000-00005 11984291

[B15] Gellman H. Chandler D. Petrasek J. Sie I. Adkins R. Waters R. (1988). Carpal tunnel syndrome in paraplegic patients. J. Bone Joint Surg. 70, 517–519. 3356717

[B16] Gutierrez D. D. Thompson L. Kemp B. Mulroy S. J. Network PTCR. (2007). The relationship of shoulder pain intensity to quality of life, physical activity, and community participation in persons with paraplegia. J. Spinal Cord Med. 30, 251. 17684891 10.1080/10790268.2007.11753933 PMC2031955

[B17] Kotajarvi B. R. Sabick M. B. An K.-N. Zhao K. D. Kaufman K. R. Basford J. R. (2004). The effect of seat position on wheelchair propulsion biomechanics. J. Rehabil. Res. Dev. 41, 403–414. 10.1682/JRRD.2003.01.0008 15543458

[B18] Learmonth Y. C. Kinnett-Hopkins D. Rice I. M. Dysterheft J. L. Motl R. W. (2015). Accelerometer output and its association with energy expenditure during manual wheelchair propulsion. Spinal Cord. 10.1038/sc.2015.33 25777327

[B19] Madeleine P. Mathiassen S. E. Arendt-Nielsen L. (2008). Changes in the degree of motor variability associated with experimental and chronic neck-shoulder pain during a standardised repetitive arm movement. Exp. Brain Res. 185, 689–698. 10.1007/s00221-007-1199-2 18030457

[B20] Medicine PVoACfSC. (2005). Preservation of upper limb function following spinal cord injury: a clinical practice guideline for health-care professionals. J. Spinal Cord Med. 28, 434. 16869091 10.1080/10790268.2005.11753844 PMC1808273

[B21] Moon Y. Jayaraman C. Hsu I. Rice I. Hsiao-Wecksler E. Sosnoff J. (2013). Variability of peak shoulder force during wheelchair propulsion in manual wheelchair users with and without shoulder pain. Clin. Biomech. 28, 967–972. 10.1016/j.clinbiomech.2013.10.004 24210512 PMC3858527

[B22] Nash M. S. Jacobs P. L. Mendez A. J. Goldberg R. B. (2001). Circuit resistance training improves the atherogenic lipid profiles of persons with chronic paraplegia. J. Spinal Cord Med. 24, 2–9. 11587430 10.1080/10790268.2001.11753548

[B23] Nyland J. Snouse S. L. Anderson M. Kelly T. Sterling J. C. (2000). Soft tissue injuries to USA paralympians at the 1996 summer games. Arch. Phys. Med. Rehabil. 81, 368–373. 10.1016/S0003-9993(00)90086-8 10724085

[B24] O’Connell D. G. Barnhart R. (1995). Improvement in wheelchair propulsion in pediatric wheelchair users through resistance training: a pilot study. Arch. Phys. Med. Rehabil. 76, 368–372. 10.1016/S0003-9993(95)80663-6 7717838

[B25] Rice I. Gagnon D. Gallagher J. Boninger M. (2010). Hand rim wheelchair propulsion training using biomechanical real-time visual feedback based on motor learning theory principles. J. Spinal Cord Med. 33, 33. 20397442 10.1080/10790268.2010.11689672 PMC2853327

[B26] Rice I. M. Pohlig R. T. Gallagher J. D. Boninger M. L. (2013). Handrim wheelchair propulsion training effect on overground propulsion using biomechanical real-time visual feedback. Arch. Phys. Med. Rehabil. 94, 256–263. 10.1016/j.apmr.2012.09.014 23022092

[B27] Robertson R. N. Boninger M. L. Cooper R. A. Shimada S. D. (1996). Pushrim forces and joint kinetics during wheelchair propulsion. Arch. Phys. Med. Rehabil. 77, 856–864. 10.1016/S0003-9993(96)90270-1 8822674

[B28] Sawatzky B. Rushton P. W. Denison I. McDonald R. (2012). Wheelchair skills training programme for children: a pilot study. Aust. Occup. Ther. J. 59, 2–9. 10.1111/j.1440-1630.2011.00964.x 22272877

[B29] Shimada S. D. Robertson R. N. Bonninger M. L. Cooper R. A. (1998). Kinematic characterization of wheelchair propulsion. J. Rehabil. Res. Dev. 35, 210–218. 9651893

[B30] Van der Woude L. De Groot G. Hollander A. van Ingen S. G. Rozendal R. (1986). Wheelchair ergonomics and physiological testing of prototypes. Ergonomics 29, 1561–1573 10.1080/00140138608967269 3102225

[B31] Vanlandewijck Y. Theisen D. Daly D. (2001). Wheelchair propulsion biomechanics. Sports Med. 31, 339–367 10.2165/00007256-200131050-00005 11347685

[B32] Vegter R. J. Lamoth C. J. De Groot S. Veeger D. H. Van der Woude L. H. (2013). Variability in bimanual wheelchair propulsion: consistency of two instrumented wheels during handrim wheelchair propulsion on a motor driven treadmill. J. Neuroeng. Rehabil. 10, 9. 10.1186/1743-0003-10-9 23360756 PMC3614450

[B33] Vegter R. J. Lamoth C. J. de Groot S. Veeger D. H. van der Woude L. H. (2014). Inter-individual differences in the initial 80 minutes of motor learning of handrim wheelchair propulsion. PLoS ONE 9:e89729. 10.1371/journal.pone.0089729 24586992 PMC3931829

[B34] Wang L. P. Hamaker E. Bergeman C. (2012). Investigating inter-individual differences in short-term intra-individual variability. Psychol. Methods 17, 567. 10.1037/a0029317 22924600 PMC3684184

[B35] Wylie E. Chakera T. (1988). Degenerative joint abnormalities in patients with paraplegia of duration greater than 20 years. Spinal Cord 26, 101–106. 10.1038/sc.1988.20 3412779

